# Osteoporosis Assessment Using Bone Density Measurement in Hounsfield Units in the Femoral Native CT Cross-Section: A Comparison with Computed Tomography X-Ray Absorptiometry of the Hip

**DOI:** 10.3390/diagnostics15081014

**Published:** 2025-04-16

**Authors:** Julian Ramin Andresen, Guido Schröder, Thomas Haider, Hans-Christof Schober, Reimer Andresen

**Affiliations:** 1 Division of Trauma Surgery, Department of Orthopaedics and Trauma Surgery, Medical University of Vienna, Währinger Gürtel 18-20, 1090 Vienna, Austria; thomas.haider@meduniwien.ac.at; 2 Department of Traumatology, Hand and Reconstructive Surgery, Rostock University Medical Center, Schillingallee 35, 18057 Rostock, Germany; guido.schroeder1@gmx.net; 3 OrthoCoast, Practice for Orthopedics and Osteology, Hufelandstraße 1, 17438 Wolgast, Germany; hcr.schober@gmx.de; 4 Institute for Diagnostic and Interventional Radiology/Neuroradiology, Westkuestenklinikum Heide, Academic Teaching Hospital of the Universities of Kiel, Luebeck und Hamburg, Esmarchstraße 50, 25746 Heide, Germany; randresen@wkk-hei.de

**Keywords:** computed tomography X-ray absorptiometry hip (CTXA-Hip), Hounsfield units (HUs), bone density measurement, bone mineral content (BMC) or bone mineral density (BMD), region of interest (ROI), osteoporosis

## Abstract

**Background/Objectives:** Bone mineral density (BMD) loss leads to osteoporosis, significantly increasing fracture risk in both the axial and peripheral skeleton. The extent to which it is possible to estimate the degree of osteoporosis in the hip by determining the density in Hounsfield Unit (HU) measurements derived from computed tomography (CT) scans and to calculate quantitative BMD and T values from the HU values should be examined. **Methods:** A total of 240 patients (mean age: 64.9 ± 13.1 years, BMI: 26.8 ± 6.8 kg/m^2^) underwent CT-based BMD assessments using CTXA-Hip. Subregions of the proximal femur, including the femoral head, femoral neck, and intertrochanteric region, were analyzed for cancellous density in HUs using circular and irregular region-of-interest (ROI) measurements. Correlations between HU values and DEXA-equivalent BMD (mg/cm^2^) and T values were computed. Predictive power for osteoporosis was evaluated using ROC curve analysis. **Results:** Cancellous bone density in the proximal femur showed a significant decline with increasing age and decreasing BMI (*p* < 0.05). The median BMD for the entire hip was 0.684 mg/cm^2^, and the median HU value for the proximal femur was 123.15. Strong correlations were observed between HU values and BMD (R^2^ = 0.904, *p* < 0.001) and T values (R^2^ = 0.911, *p* < 0.001). A T value of −2.5 corresponded to an HU value of 95.79 in the entire femur. ROC analysis demonstrated high sensitivity (0.92) and specificity (0.93) for HU-based osteoporosis prediction. **Conclusions:** HU measurements provide a reliable method for estimating BMD and T values in the proximal femur, offering a valuable diagnostic tool for osteoporosis. The highest predictive accuracy was achieved when using an irregular ROI from the entire proximal femoral region.

## 1. Introduction

An increasing loss of bone mineral density (BMD) leads to osteoporosis and an increased risk of fractures in the axial skeleton [1,2,3] and peripheral areas—in this case, with an accumulation of fractures of the hip, distal radius, and proximal humerus [4,5]. The number of hip fractures worldwide was calculated to be around 1.7 million in 1990 and is estimated to rise to around 6.3 million in 2050 [6]. According to an evaluation of hospital diagnosis statistics for Germany from 2004, Icks et al. estimated 116,000 people with at least one hip fracture per year [7]. Fractures in the femoral neck and the pertrochanteric region showed an increase of more than 20% in Germany from 2009 to 2019; for 2019, the incidence of femoral neck fractures was 120.2 per 100,000, and that of pertrochanteric fractures was 108.7 per 100,000 inhabitants [8], while the number of surgical interventions is currently 135,000 per year. In patients with hip fractures, silent vertebral compression fractures are frequently observed [5]; in this context, proximal femur fractures, as indicator fractures, represent an imminent risk of further fractures [9]. For osteoporotic hip fractures, there is a mortality rate of 20 to 40% in the first year after the fracture event [10,11]. In a study conducted in Spain, men were found to have a higher mortality rate (43%) than women (30%) during the first year after the fracture event [12]. Only 30 to 40% of patients with osteoporotic hip fractures regain their previous mobility [13]. Patients requiring care in nursing homes benefit from orthogeriatric treatment, experiencing improved functional outcomes, a lower risk of subsequent fractures, and reduced mortality rates [14,15]. Key risk factors for osteoporosis and, in particular, hip-related fractures include advanced age; low bone mineral density (BMD); type I and type II diabetes mellitus; a history of hip fracture, pelvic fracture, vertebral fracture, or humeral fracture; and conditions such as Parkinson’s disease, Alzheimer’s disease, multiple sclerosis, and epilepsy. Additional risk factors include systemic glucocorticosteroid therapy, rheumatoid arthritis, low BMI, vitamin D deficiency, female sex, hyponatremia, and excessive nicotine and alcohol consumption [16,17,18].

The gold standard for assessing bone density and diagnosing osteoporosis is dual-energy X-ray absorptiometry (DEXA) [19]. According to this method, a T value between −1.5 and −2.5 indicates osteopenia, while a score below −2.5 defines osteoporosis [20]. This classification applies equally to both the spine and the hip. As an alternative, the CTXA-Hip method provides DEXA-equivalent values for the hip, allowing for reliable osteoporosis assessment in this region [21,22,23,24].

To what extent osteoporosis severity in the proximal femur can be estimated using trabecular bone density measurements in Hounsfield Units (HUs) from native CT images and whether quantitative BMD and T values can be derived from HU values was evaluated in comparison with CTXA-derived values. The influences of different regions of interest (ROIs) in terms of shape and size were also taken into account. This topic is becoming increasingly important as more and more CT scanners are available compared to DXA devices and CT examinations of the pelvis and hip region are increasingly available for various clinical questions.

## 2. Materials and Methods

### 2.1. Study Design and Ethical Approval

This multicenter, retrospective study was conducted following approval from the responsible ethics committee of the university medical center (Approval ID: D 471/24). The study adhered to national regulations and complied with the principles of the Declaration of Helsinki.

### 2.2. Study Population

A total of 240 patients (mean age: 64.9 ± 13.1 years; BMI: 26.8 ± 6.8 kg/m^2^) were included in the study, of whom 40 were male (mean age: 60.0 ± 14.3 years; BMI: 28.4 ± 5.7 kg/m^2^) and 200 were female (mean age: 65.8 ± 12.7 years; BMI: 26.5 ± 7.0 kg/m^2^) (Table 1). The study aimed to assess the presence of osteoporosis in these patients. Participants were referred from outpatient clinics specializing in bariatric surgery, gynecology, geriatrics, neurosurgery, orthopedics, and trauma surgery.

Patients with a history of total hip arthroplasty were excluded due to potential metal artifacts affecting imaging results. Additionally, individuals with known malignancies or those found to have metastatic bone lesions during the examination were excluded from the study.

### 2.3. Diagnostic Procedures

Bone mineral density (BMD) and T values were quantitatively assessed using computed tomography X-ray absorptiometry of the hip (CTXA-Hip), which provides dual-energy X-ray absorptiometry (DEXA)-equivalent values (GE-Revolution EVO/64 line CT with Mindways software, CTXA-Hip BMD Application Module, Version 4.2.3, Austin, TX, USA).

**Figure 1:**
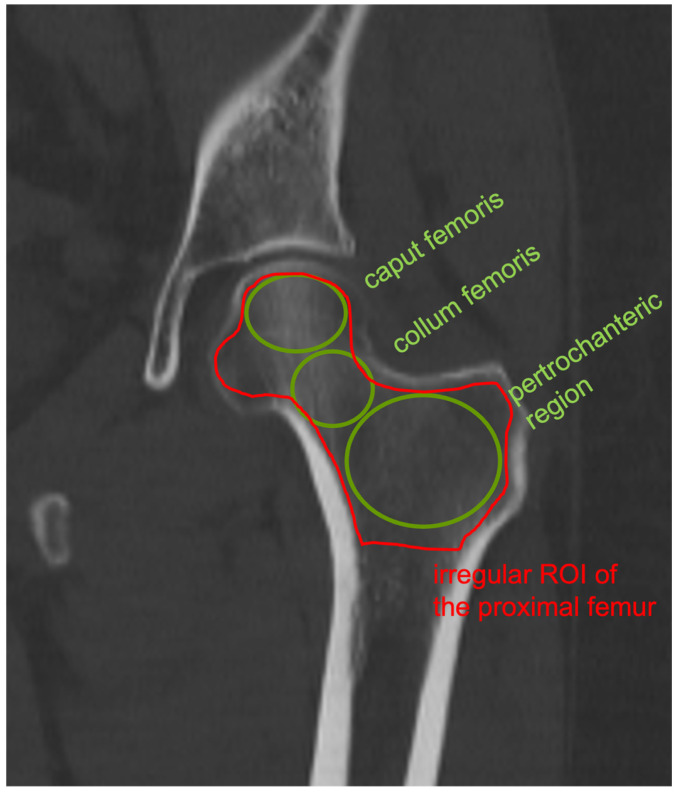
Coronal CT image of the left hip with correspondingly marked ROIs in the proximal femur.

For the entire proximal femur, trabecular density was determined in Hounsfield units (HUs) using circular ROIs in the femoral head, femoral neck, and pertrochanteric region (Figure 1). The size of the individual ROIs was chosen so that they had an approximate distance of 1 mm to the cortical shell. Additionally, an irregularly shaped ROI covering the entire proximal femur was applied, and the mean values of the summed individual ROIs were considered for further analysis. In order to obtain the largest possible and most reproducible coronal surface of the proximal femur, this was defined with a suitable cutting plane in the axial CT section and multiple planar reconstruction.

All CT scans were performed at a tube voltage of 120 kV, with a slice thickness of 2 mm and a window setting of C = 400/W = 1600.

### 2.4. Statistical Analysis

Statistical analysis was conducted using SPSS version 23.0 (SPSS Inc., Armonk, NY, USA). Quantitative variables are presented as mean (M) ± standard deviation (SD) and the number of observations (*n*) for parametric tests, while non-parametric variables are expressed as median with first and third quartiles (Q1–Q3).

Spearman’s correlation coefficient (r) was used to assess relationships between variables. A regression analysis was performed to estimate CTXA values using a generalized estimating equation. Statistical significance was set at *p* < 0.05.

Effect sizes were calculated according to Cohen’s guidelines: values below 0.5 were considered small, those between 0.5 and 0.8 were considered moderate, and those above 0.8 were considered large.

A receiver operating characteristic (ROC) curve analysis was performed to evaluate the predictive power of HU values for osteoporosis across the entire proximal femur, as well as within individual regions (femoral head, femoral neck, and intertrochanteric region) and for the combined ROI dataset. To compare the AUC values of different ROI-based models, DeLong’s test was applied to determine whether differences in diagnostic performance were statistically significant.

## 3. Results

**Figure 2:**
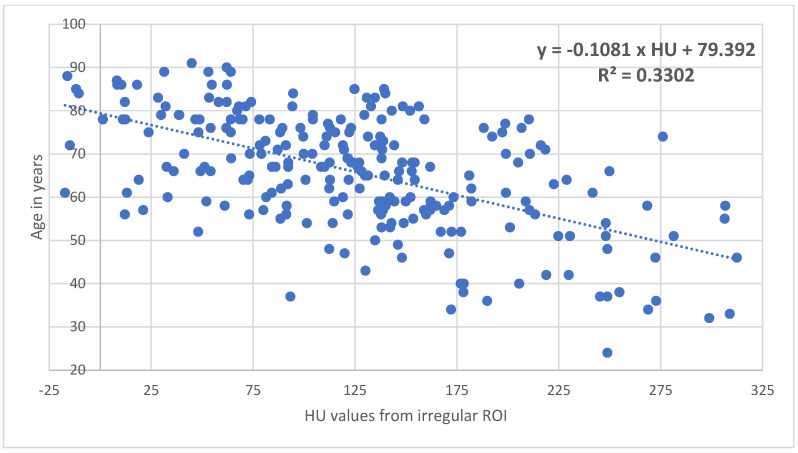
In the proximal femur, there is a significant (*p* < 0.05) decrease in cancellous density in HUs with increasing patient age.

**Figure 3:**
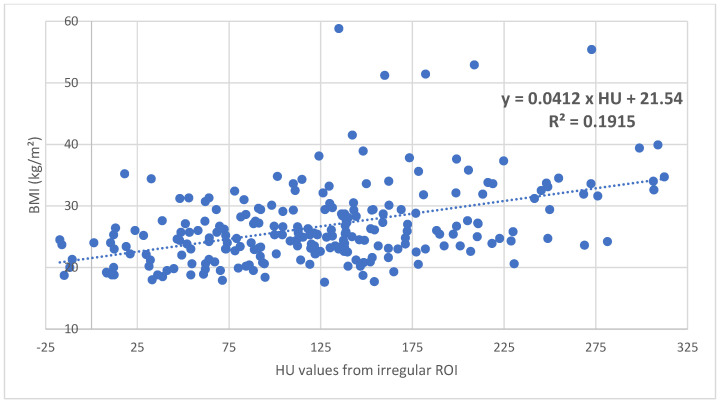
In the proximal femur, there is a significant (*p* < 0.05) increase in cancellous density in HUs with increasing BMI.

Patients with increasing age and decreasing BMI showed significantly (*p* < 0.001) decreasing BMD and HU values (Figure 2 and Figure 3).

**Figure 4:**
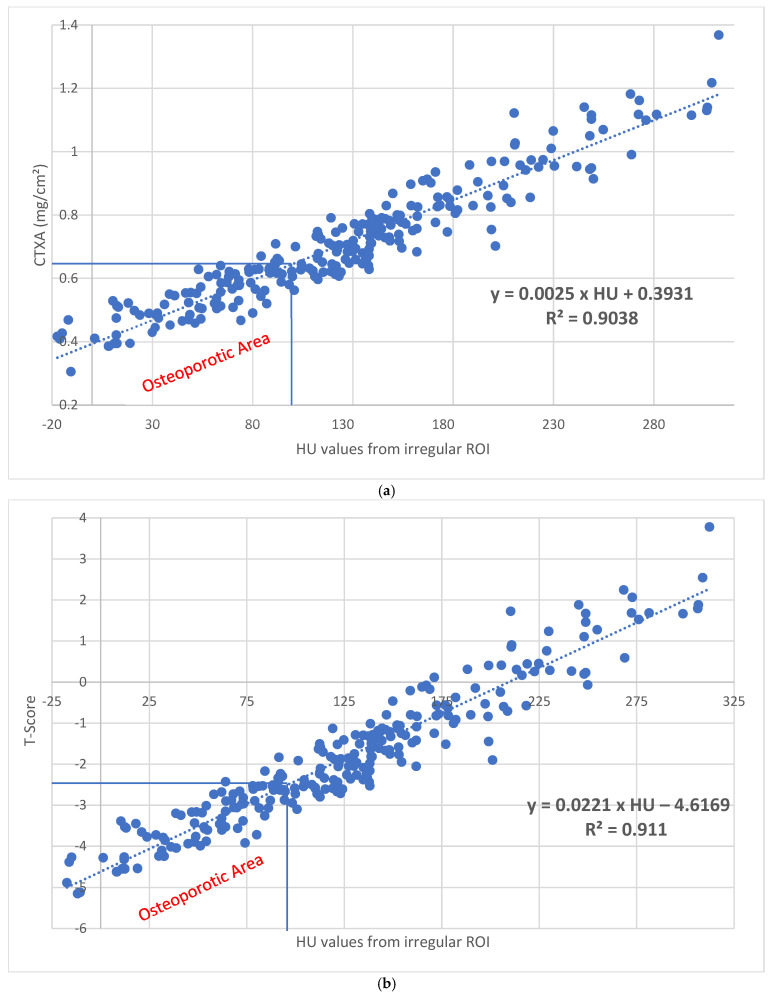
(**a**) With a correlation of R^2^ = 0.9038 (*p* < 0.001), the following formula can be used: X_ctxa, total irregular area_ = 0.393 + 0.0025 × HU quantitative values in mg/cm^2^. A value of 95.79 HU results in 0.633 mg/cm^2^, which represents the threshold for osteoporosis. (**b**) With a correlation of R^2^ = 0.911 (*p* < 0.001) for the entire hip, corresponding T values can be calculated using the following formula: X_total, irregular region_ = −4.6169 + 0.0221 × HU. A value of 95.79 HU results in a T value of −2.5, which represents the threshold for osteoporosis.

For the entire hip (Table 2), the median BMD was 0.684 (0.306–1.368) mg/cm^2^, and the median HU value for the entire proximal femur region (data from the irregular-surface ROI) was 123.15 (−17.3–312.4). With a correlation of R^2^ = 0.904 (*p* < 0.001), the following formula can be used: $X_{ctxa}=0.393+0.0025\times HU$. Quantitative values in mg/cm^2^ can be calculated from the HU values (Figure 4a).

The median T value (Table 2) was −2.06 (−5.15–3.78). With a correlation of R^2^ = 0.911 (*p* < 0.001), corresponding T values can be calculated from the HU values using the following formula: ${Xt}_{total,irregular-area-ROI}=-4.617+0.0221\times HU$ (Figure 4b). Here, 95.79 HU corresponds to a T value of −2.5.

**Figure 5:**
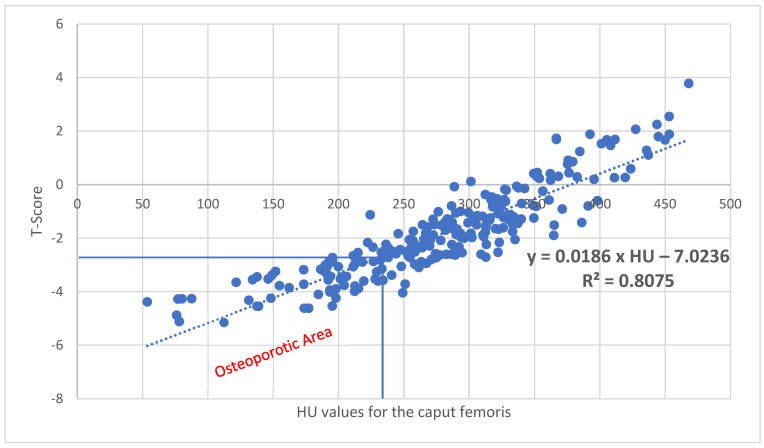
With a correlation of R^2^ = 0.8075 (*p* < 0.001) for the caput femoris, corresponding T values can be calculated using the following formula: Xt_Caput femoris_ = −7.0236 + 0.0186 × HU. A value of 243.2 HU results in a T value of −2.5, which represents the threshold for osteoporosis.

With a correlation of R^2^ = 0.8075 (*p* < 0.001) for the caput femoris, corresponding T values can be calculated using the following formula: ${Xt}_{Caput\;femoris}=-7.0236+0.0186\times HU$ (Figure 5). Here, 243.2 HU corresponds to a T value of −2.5.

**Figure 6:**
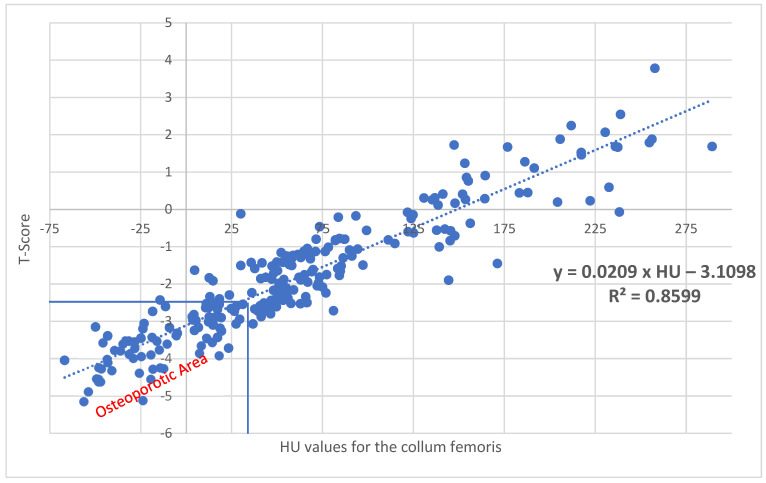
With a correlation of R^2^ = 0.8599 (*p* < 0.001) for the collum femoris, corresponding T values can be calculated using the following formula: Xt_Collum femoris_ = −3.1098 + 0.0209 × HU. A value of 29.2 HU results in a T value of −2.5, which represents the threshold for osteoporosis.

With a correlation of R^2^ = 0.8599 (*p* < 0.001) for the collum femoris, corresponding T values can be calculated using the following formula: ${Xt}_{Collum\;femoris}=-3.1098+0.0209\times HU$ (Figure 6). Here, 29.2 HU corresponds to a T value of −2.5.

**Figure 7:**
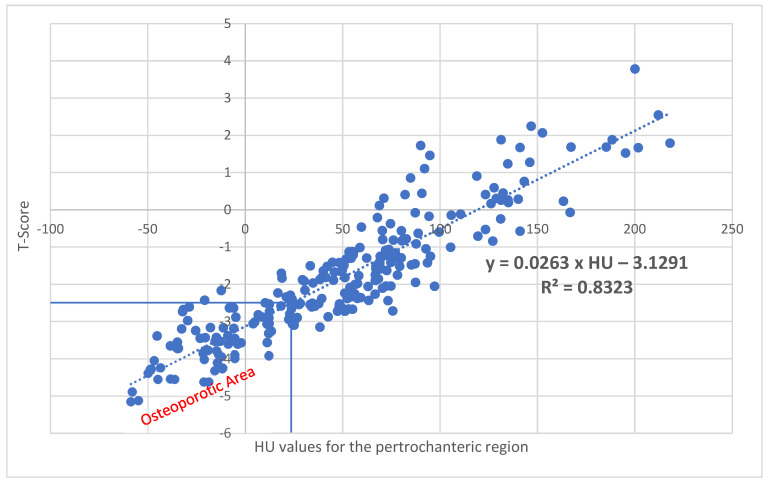
With a correlation of R^2^ = 0.8323 (*p* < 0.001) for the pertrochanteric region, corresponding T values can be calculated using the following formula: Xt_pertrochanteric region_ = −3.1291 + 0.0263 × HU. A value of 23.9 HU results in a T value of −2.5, which represents the threshold for osteoporosis.

With a correlation of R^2^ = 0.8323 (*p* < 0.001) for the pertrochanteric region, corresponding T values can be calculated using the following formula: ${Xt}_{pertrochanteric\;region}=-3.1291+0.0263\times HU$ (Figure 7). Here, 23.9 HU corresponds to a T value of −2.5.

**Figure 8:**
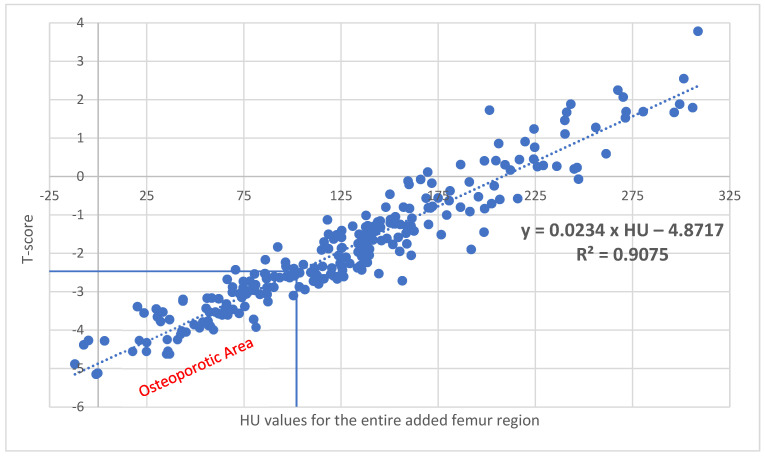
With a correlation of R^2^ = 0.9075 (*p* < 0.001) for the entire added femur region, corresponding T values can be calculated using the following formula: Xt_total, addition area_ = −4.8717 + 0.0234 × HU. A value of 101.4 HU results in a T value of −2.5, which represents the threshold for osteoporosis.

With a correlation of R^2^ = 0.9075 (*p* < 0.001) for the sum of the added ROI data, corresponding T values can be calculated using the following formula: ${Xt}_{total}=-4.8717+0.0234\times HU$ (Figure 8). Here, 101.4 HU corresponds to a T value of −2.5.

When comparing the median data from the irregular-area ROI with the data from the additive ROIs, the data from the irregular-area ROI are lower by 4.95 HU (3.86%) (Table 3), and with a T value of −2.5, they are lower by 5.61 HU (5.53%) (Figure 4b and Figure 8).

**Figure 9:**
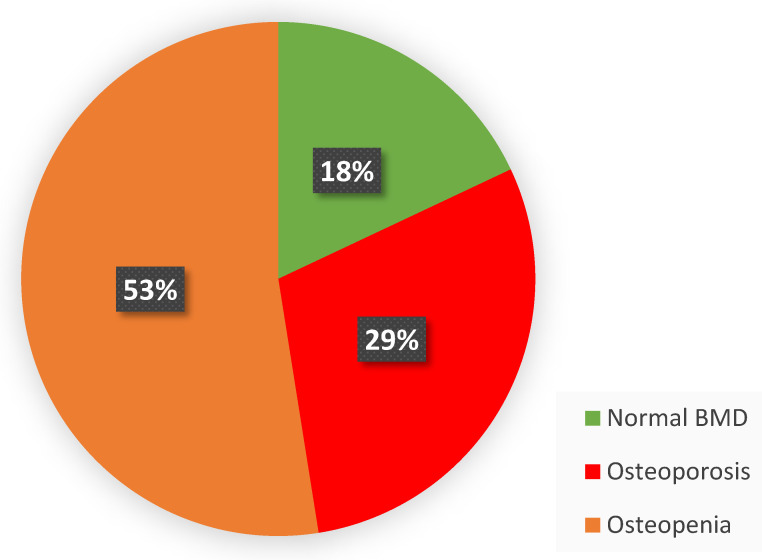
Based on the T value, the graph shows the number of patients with osteoporosis, osteopenia, and normative BMD. The threshold T value were used for the groupings, as defined in the DVO guideline on osteoporosis [20].

Taking the T value into account, Figure 9 shows the percentage distribution of the patient collective under normal, osteopenia, and osteoporosis conditions.

**Figure 10:**
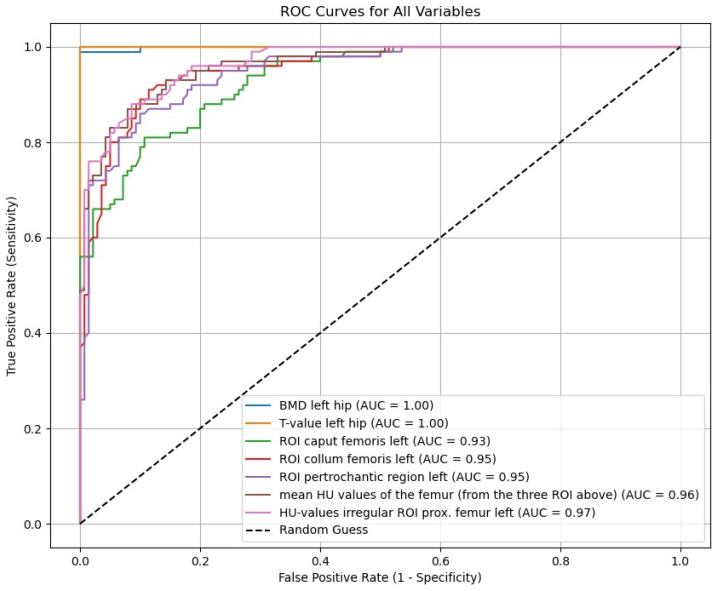
Using ROC curve analysis, it can be shown that HU values of the entire proximal femur region in the osteoporosis assessment are in high agreement with the BMD (mg/cm^2^) and T values from CTXA. Here, the irregular-area ROI with an AUC = 0.97 shows the highest predictive power for osteoporosis, followed by the added-area ROI with an AUC = 0.96, the ROIs in the pertrochanteric region and the collum femoris with an AUC = 0.95 each, and the ROI of the caput femoris with an AUC = 0.93.

Using ROC curve analysis, it can be shown that HU values of the entire proximal femur region (data from the irregular-area ROI) are in high agreement (AUC = 0.97) with the BMD (mg/cm^2^) (sensitivity = 0.92; specificity = 0.93) and the T values (sensitivity = 0.92; specificity = 0.93) at an osteoporosis threshold of 95.79 HU, and no significant difference is found with *p* = 0.395. This results in an effect size of 0.89. An osteoporosis estimate taking into account the individual regions and the sum of the added ROI data is significant (*p* < 0.001) for different threshold values but somewhat less accurate (Figure 10).

## 4. Discussion

In comparison with the DXA-hip, the determination of cancellous density in HUs in the coronal CT section of the femur is free of degenerative overlapping and does not require the adding up of the cortical bone. Using an individually inserted ROI that ends as close as possible to the cortical bone, it is possible to selectively determine the cancellous density in the caput femoris, collum femoris, and pertrochanteric region (Figure 1). The total cancellous density of the proximal femur in HUs shows a significant decrease with increasing patient age (Figure 2) and decreasing BMI (Figure 3), which underpins advanced patient age [16,17,18] and low BMI values as risk factors for osteoporosis [17,18]. Using ROC curve analysis, it can be shown that HU values of the entire proximal femur region (data from the irregular surface ROI) are highly correlated with the BMD (mg/cm^2^) (sensitivity = 0.92. Specificity = 0.92; specificity = 0.92) and T values (sensitivity = 0.92; specificity = 0.93) at an osteoporosis threshold of 95.79 HU, while no significant difference was found with *p* = 0.395. The determination of the HU values over the entire proximal femur region shows the highest significance (AUC = 0.97) for the prediction of osteoporosis in comparison with the density values for the caput femoris, collum femoris, and pertrochanteric region (Figure 10). This is also plausible, as the total trabecular area of the proximal femur is completely recorded; thus, the possible inaccuracies of individual ROIs are compensated for.

Zhao et al. [25] found osteoporosis threshold values with high predictive power of 192.23 HU for the left proximal femur and 188.71 HU for the right proximal femur, clearly exceeding our values, possibly due to an abrasion too close to the cortical bone. Nevertheless, the entire proximal femur region shows a positive correlation between the HU and BMD values with an R^2^ = 0.826, which is even more pronounced in our case with R^2^ = 0.904 (Figure 4a).

For the cancellous density in the left caput femoris, in our study, an osteoporosis threshold of 243.2 HU was found, which corresponds to a T value of −2.5 (Figure 5). Lee et al. [26] found a threshold value for osteoporosis of 296.15 HU, which is significantly higher than our values due to a smaller ROI being located centrally at the highest cancellous density of the caput femoris. In comparison to the DXA measurement, Kilinc et al. [27] also found a strong correlation with the HU values of the caput femoris, with the osteoporosis threshold value being 198 HU for the left hip and 204 HU for the right hip. In comparison to our data, the authors arrived at significantly lower osteoporosis threshold values, which can be explained by differences in ROI size and a greater distance to the cortical bone in our study. It is also possible that measured values from an axial sectional plane are not directly comparable with those from a coronal plane.

Fan et al. [28] were able to show that with HU values of the caput femoris < 124.85, the risk of implant failure after treatment of an intertrochanteric fracture with an intramedullary nail was significantly increased. These values are clearly in the osteoporotic range, whereas the osteoporosis threshold in our collective was 243.2 HU.

Overall, differences in the specified limit values are caused by non-comparable patient collectives [3] and different technical parameters, such as kV levels of the CT tube voltage, window settings, slice thicknesses, and ROI sizes, as well as shapes with undefined borders with the cortical bone and centering of the ROIs and CT slice planes [25,26,27,28,29,30]. Automatic contour finding programs, which are easy to implement, could further optimize the measurements. Standardization is urgently needed for comparability of literature data. If this is taken into account, osteoporosis estimation from existing native CT scans is easily possible without extensive examinations. Such estimations can then be used as a surrogate parameter for fracture risk assessment [30] and, if necessary, drug therapy planning.

In general, the determination of cancellous density in HUs for the assessment of osteoporosis and fracture risk assessment in CT cross-sectional images is becoming increasingly important in everyday clinical practice, with valid results being found for the hip region [25,26,27,28,31,32,33,34] and on the axial skeleton [2,3,35,36,37,38].

Targeted density measurements in the dens axis [39] and the proximal humerus [40] also appear possible and helpful for local structural analysis and fracture risk assessment. If CT data are already available, they can be used, eliminating the need for further radiation exposure and additional costs.

Further research is needed to refine HU-based threshold values across different patient populations [3,38] and to explore automated methods for standardized ROI selection. Additionally, incorporating HU-based bone density assessments into fracture risk prediction models may help optimize individualized treatment plans and prevent osteoporotic fractures in high-risk patients.

## 5. Limitations and Strengths

This was a retrospective study. The patient cohort was not based on a random selection from the general population but on referrals from specialized outpatient clinics, which could have led to a selection bias, possibly limiting the generalizability of the results. In contrast, there was a wide range of patient age and BMI in the patient collective, which should correspond to a broad section of the general population.

The ROI size and placement introduced by hand are subject to a certain degree of variability. The HU values were compared with CTXA data and not with the gold-standard DXA. Patients with hip fractures were not included in the study.

## 6. Conclusions

This study demonstrates that trabecular bone density measurements in Hounsfield units (HUs) from native CT scans provide a reliable method for assessing osteoporosis in the proximal femur. The strong correlation between HU values and BMD measurements obtained through CTXA-Hip suggests that HU-based assessments can serve as an effective surrogate for the diagnosis of osteoporosis and estimation of fracture risk.

Our findings indicate that an HU threshold of 95.79 in the entire proximal femoral region, based on the irregular ROI measurement, corresponds to a T value of −2.5, the standard diagnostic threshold for osteoporosis. This threshold showed high predictive accuracy, with strong agreement between HU, CTXA-BMD, and T values. Compared to regional ROI measurements, the irregular ROI method encompassing the entire proximal femur provided the most precise correlation with standard BMD assessments.

The ability to derive BMD and T values directly from routine CT scans without additional imaging offers significant clinical advantages, particularly in opportunistic screening scenarios. Given the widespread availability of CT imaging, integrating HU-based osteoporosis assessment into clinical practice could enhance early detection and intervention strategies, ultimately improving patient outcomes.

In addition, the data collection and standardized evaluation of HU values from different CT scanners with different examination protocols and heterogeneous examination collectives through the use of AI-based image analysis, including the implementation of automated segmentation techniques based on machine learning algorithms to reduce inter- and intra-operator variability, seems promising to further increase reproducibility and precision and, thus, enable the clinical applicability of HU-based osteoporosis diagnostics and fracture risk determination on a broad scale, which can be made possible for clinicians through available open-source tools [41].

## Figures and Tables

**Table 1:** Patient description by gender, age, and BMI.

Characterization of the Total Sample			
	Patients (*n* = 240)	Men (*n* = 40)	Women (*n* = 200)
Age	⌀ 65.9	⌀ 68.5	⌀ 65.3
(in years)	(min.: 24; max.: 91)	(min.: 42; max.: 84)	(min.: 24; max.: 91)
BMI	⌀ 26.7	⌀ 24.9	⌀ 27.1
(kg/m^2^)	(min.: 17.6; max.: 60.6)	(min.: 17.6; max.: 33.8)	(min.: 17.7; max.: 60.6)

**Table 2:** CTXA and T values of the entire hip.

CTXA and T Values of the Hip				
	*n*	Min	Max	Median
CTXA (mg/cm^2^)	240	0.3055	1.368	0.684
T-value	240	−5.15	3.78	−2.06

**Table 3:** HU values from the different ROIs of the proximal femur.

HU Values of the Different ROIs				
ROI	*n*	Min	Max	Median
Caput femoris	240	53.6	468.1	281.65
Collum femoris	240	−66.8	289.3	51.75
Pertrochanteric region	240	−58.8	218.1	50.9
Average values from the upper three ROIs	240	−24	325.17	128.1
Irregular-surface ROI of the prox. femur	240	−17.3	312.4	123.15

## Data Availability

The raw data supporting the conclusions of this article will be made available by the authors upon reasonable request.

## Abbreviations

The following abbreviations are used in this manuscript:

AUC	Area under the curve
BMD	Bone mineral density
BMI	Body mass index
CTXA	Computed tomography X-ray absorptiometry
DEXA	Dual-energy X-ray absorptiometry
Fig.	Figure
GE	General Electric
HU	Hounsfield unit
MA	Mean age
*n*	Number
OVF	Osteoporotic vertebral fracture
Q1-Q3	First to third quartiles
ROC	Receiver operating characteristic
ROI	Region of interest
SD	Standard deviation
Tab.	Table
